# Research application of session-RPE in monitoring the training load of elite endurance athletes

**DOI:** 10.3389/fnins.2024.1341972

**Published:** 2024-05-23

**Authors:** Shengjie Yang, Yiquan Yin, Zhaoyi Qiu, Qingjun Meng

**Affiliations:** Department of China Skiing and Ice Sport College, Beijing Sport University, Beijing, China

**Keywords:** cross-country skiing, training impulse, RPE, session-RPE, training load

## Abstract

**Purpose:**

TRIMP and sRPE are both representative indicators of training load(TL), and the correlation between two has been widely demonstrated across various sports. The aim of this study was to investigate the reliability of sRPE-TRIMP correlation across different intensities/duration of training in cross-country skiing, and whether sRPE can serve as an validity supplement to TRIMP data in cases of lost heart rate data.

**Method:**

10 athletes were used as the experimental objects. The intensity, duration and RPE of 273 different types of training sessions were collected, and statistical methods were used for data analysis.

**Results:**

1. There was a significant correlation between sRPE and TRIMP (*r* = 0.68, *p* < 0.05), but the correlation differs among the LIT, MIT and HIT groups (*r* = 0.70, 0.46, *r* = 0.31, *p* < 0.05) 2. sRPE-TRIMP correlation among three different time duration in the LIT group (0-60 min, 60-120 min and 120-180 min), are all highly significant (*r* = 0.70, 0.67, 0.69, *p* < 0.05) and the LRsRPE-TRIMP of 3 duration have no significant differences (chow test, *p* > 0.05). 3. The difference in actual training duration between samples was the main reason for the difference in the application effect of sRPE, because the actual training duration ratio of LIT was 89.7 ± 16.4%; MIT, 98.5 ± 6.2%; and HIT, 94.4 ± 13.5%.

**Conclusion:**

1. The linear relationship between sRPE and TRIMP (LRsRPE-TRIMP) is more significant in LIT compared to that in MIT and HIT. 2. Variations in the duration of LIT sessions do not affect the consistency of the relationship between sRPE and TRIMP. 3. Discrepancies between actual and planned training durations directly impact the significance of the LRsRPE-TRIMP.

## 1 Introduction

The study of training load is a central issue in the field of sports training (Giordano et al., 2017). Load stimulation results from the interaction between duration, training intensity, activity form, frequency, and training recovery (Seiler and Tønnessen, 2009; Laursen, 2010). Monitoring the load is the premise to ensure the scientific arrangement of training. During exercise, external load input elicits a response in internal load, leading to adaptations in athletic performance. Internal load is complex, encompassing both physiological and psychological responses. In sports science, there are numerous indicators of internal and external load indicators, and the validity of a single indicator depends on the specific characteristics of sport (Impellizzeri et al., 2019).

Both HR and RPE are considered to be internal training load metrics (Impellizzeri et al., 2019). The former method is to use incremental load experiments to calibrate the heart rate zone, record the TL in each intensity zone according to the heart rate during training, and then perform TRIMP calculation. The later is calculating sRPE based on the combination of RPE and training session duration, which is more simple, convenient, non-invasive, and does not require specific equipment (Gabbett et al., 2017), and the load can be assessed at any time.

Due to its nearly linear relationship with VO2 across a broad spectrum of steady-state submaximal exercise intensities, heart rate is widely utilized in load monitoring for endurance activities, with TRIMP (Training Impulse) commonly calculated based on heart rate intensity (Wallace et al., 2013). Meanwhile, sRPE (session rating of perceived exertion) serves as another widely applicable method for load monitoring, with studies often investigating the correlation between these two internal load indicators. Therefore, many studies validating the reliability of sRPE have used significant correlation with TRIMP as the criterion, and the sRPE-TRIMP correlation has been verified in many studies (Haddad et al., 2017). Most studies mainly focus on sports with varying intensity like ball sports, and less research has been done on endurance training (Haddad et al., 2017). Furthermore, in the limited endurance training studies, there was no examination of the classification of sRPE predictive effects of different training intensities (low-intensity training [LIT], medium-intensity training [MIT], and high-intensity training [HIT]). Most studies have verified the significance between sRPE and TRIMP under one condition. In endurance sports like cross-country skiing, training load is typically calculated based on heart rate. However, issues such as heart rate loss or device malfunction are common. Using sRPE to estimate TRIMP can address this problem. Moreover, the conversion relationship between sRPE and TRIMP may vary across training sessions of different intensities, then the consistency of the linear relationship (LRsRPE-TRIMP) between session-RPE and TRIMP in different intensity training courses is very important. This is an important content of this study.

The duration of the training session is also an important factor to consider. Cross-country skiing has an extremely long training duration, and a single training session often exceeds 3 h. relevant research on continuous training believes that the length of training may affect RPE, and the excessively long training duration may cause RPE to be overestimated (Green et al., 2009).

Additionally, in cyclic movement endurance sports like cross-country skiing, interval training is pre-planned, where athletes strive to maintain a predetermined high-intensity zone. However, fluctuations in fatigue and athlete’s emotional state can result in significant differences in ‘Time in zone,’ even when using the same training plan and exertion level. It is necessary to pay attention to whether this difference exists, and to explore whether the difference between actual and planned training duration will affect the validity of sRPE measurement.

The aim of this study is to examine the significance of LRsRPE-TRIMP across different intensities, and to assess its consistency across different training duration.

Research hypothesis:

*Assumption* 1: In different intensity training, significant linear regression relationships can be established between sRPE and TRIMP, but there is a lack of consistency between different linear regressions.

*Assumption* 2: In continuous low-intensity training, there is a lack of consistency between the linear regressions established by training samples with different training durations.

*Assumption* 3: The difference between the planned training duration and the actual training duration is the main factor affecting the LRsRPE-TRIMP.

## 2 Methods

### 2.1 Research object

This study was carried out during a six-month (7–12 month) team observation in the 2022–2023 season, in which the general physical preparation period (GP_2_) was from July to November, and the specific preparation period (SP) was from November to December. We included 10 athletes from the national cross-country skiing training team as the research participants (males = 5, age 18.3 ± 1.5 years; females = 5, age 17.3 ± 1.4 years; Table 1), and included the training parameters of athletes from 273 training sessions as research samples (Table 2). This study was approved by the university’s ethics committee; participants were informed of the objectives, possible risks, and benefits of the study.

**Table 1:** Athlete basic indicators.

Index	Male (*n* = 5)	Female (*n* = 5)
Age(years)	18.3 ± 1.5 (16–21)	17.3 ± 1.4 (15–20)
Height (cm)	182.9 ± 6.3 (173–190)	167.9 ± 5.9 (160–180)
Weight (kg)	69.6 ± 5.4 (62–78)	57.3 ± 3.7 (51–65)
VO_2max_ (ml·kg^−1^ min^−1^)	72.3 ± 6.1 (63.2–78.1)	58.9 ± 4.3 (52.5–72.2)

**Table 2:** Session samples collection.

Sample training session		Sample size	Session duration(h)	sRPE	TRIMP
Endurance training	---	273			
LIT	0-60 min	27	47.8 ± 12.4	138.7 ± 63.8	51.1 ± 13.2
	60-120 min	123	92.1 ± 18.1	288.5 ± 121.6	94.4 ± 32.7
	120-180 min	51	151.2 ± 19.7	581.6 ± 226.5	152.8 ± 43.9
MIT	---	32	105.1 ± 7.1	466.9 ± 213.8	143.5 ± 29.7
HIT	---	40	101.4 ± 16.9	483.3 ± 180.8	134.4 ± 31.3

The athlete were all fully informed of the nature of the study, which was pre-approved by the Regional Ethical Review Board in China (2022013H), before providing their written consent to participate.(Attachment: Informed consent form+ethical certificate).

### 2.2 Overall study design

This study is an observational study of 10 cross-country skiers with three different intensity training courses (HIT, MIT, LIT), The aim is to explore the reliability and consistency of sRPE across three types of TL when TRIMP serves as the standard. The reliability judgment depends on the significance of the linear regression test between the sample sRPE and TRIMP, and the consistency judgment depends on the difference test of the slope of the sRPE and TRIMP linear regression equation between different samples.

The study is divided into two parts. In the first part of the study, the threshold heart rate of the observed subject is determined through incremental load experiments, so that the heart rate monitoring equipment can be used to measure the subject’s TRIMP in subsequent observations; the second part is Observational research, collecting subjects’ heart rate and sRPE during endurance training of 3 intensity zone, 273 LIT, 32MIT and 40HIT were collected in this part (Table 2).

Selection criteria for participating athletes: 5 male and 5 female cross-country skiers will be randomly selected from the Chinese national cross-country skiing training team. All participants are at or above the national level of sports.

### 2.3 Flow chart

**Figure 1:**
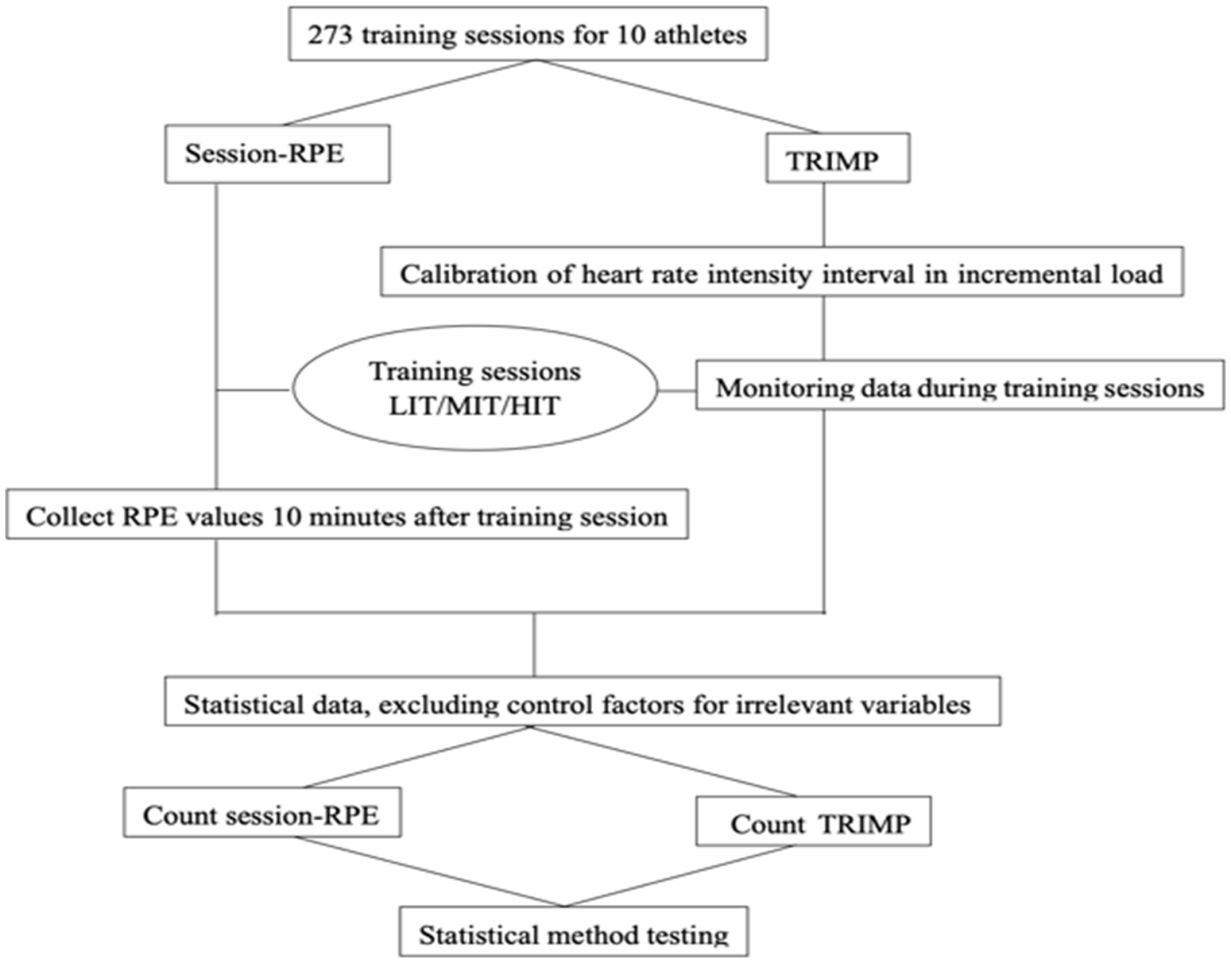
Flow chart.

Using the average data of all surveyed athletes in each training session as a sample (Figure 1).

### 2.4 Threshold heart rate calibration experiment

We used a Cortex Metalyz spirometry tester (3B, Germany) and a treadmill with incremental speed and incline for incremental load testing, and a PolarV800 heart rate belt to capture athlete heart rate data.

The primary parameter measured was VO2max expressed in milliliters per kilogram per minute (ml·kg-1·min-1). Gas analyzers for oxygen (O2) and carbon dioxide (CO2) were calibrated for every test with a specific gas concentration (O2 14.96% and CO2 5.12% and NO2 rest) according to the manufacturer’s recommendation.

The stage of test procedure was conducted on a treadmill with the initial speed 2.75 km/h and 10% of slope angle. In each 3 min interval, the slope angle increased 5% with the speed escalation 2.2 km/h that was conducted until the athlete reached a fatigue condition. The test protocol was adopted from Bruce’s maximal oxygen uptake test method (Ingjer, 2007).

Lactate threshold test method: Immediately after the end of each incremental stress test, a fingertip capillary blood sample (25 mL) was collected and instantly analyzed using an electroenzymatic technique (YSI 1500 Sport, Yellow Springs Instruments, Yellow Springs, OH). Before each test, 0, 5, 15, and 30 mmol/L standard lactic acid solution analyzers were used for calibration. To determine the 2.5 mmol/L and 4 mmol/L levels (Faude et al., 2009), and the heart rate was calibrated accordingly to determine the heart rate intensity zone of each athlete (Table 3; Sylta, 2017).

**Table 3:** Intensity scale.

5-zone model	3-zone model	Binary model	HR (%max)	Lactate (mM)
5	3/HIT	HIT	92–97	6–10
4			87–92	4–6
3	2/MIT		82–87	2.5–4
2	1/LIT	LIT	72–82	1.5–2.5
1			55–72	0.8–1.5

### 2.5 Collection process

#### 2.5.1 Heart rate data collection

**Figure 2:**
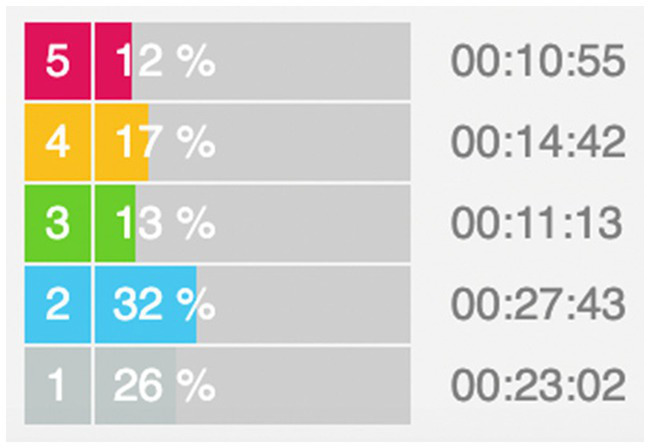
Statistical diagram of heart rate zones. Note: gray and blue are LIT zones, green are MIT zones, yellow and red are HIT zones.

We measured each athlete’s maximum heart rate through a treadmill incremental load test, calibrated the heart rate intensity zone, inputted the athlete’s heart rate interval into the polar flow, and included the three intensities of LIT, MIT, and HIT. Gray and blue zones corresponded to LIT zones, green zones corresponded to the MIT zone, and yellow and red zones corresponded to HIT zones (Figure 2). The time in zone (TIZ) method was used to collect and summarize the data (Sylta et al., 2014).

#### 2.5.2 sRPE data collection

We initially introduced each athlete to the RPE scale and the rules for its use. The scale used in this study was proposed by Foster et al. (2001). This study used the Foster 10 scale, which has been proven to have the highest correlation with the internal load of heart rate characterization (Foster et al., 2001). We collected athlete RPE through electronic devices (Roos et al., 2018), and set 11 options in the WeChat applet according to Foster’s RPE scale (0–10 point system; Table 4). The athletes were reminded 30 min after training to answer a questionnaire (Foster et al., 2001) with the question, “How did your training feel?”

**Table 4:** Foster’s modified RPE (0–10) scale.

Rating	Descriptor
0	Rest
1	Very, Very Easy
2	Easy
3	Moderate
4	Somewhat Hard
5	Hard
6	--
7	Very Hard
8	--
9	--
10	Maximal

### 2.6 Training data processing

#### 2.6.1 Control factors that exclude irrelevant variables

We excluded the course samples with missing data due to training interruptions or signal instability.

**Figure 3:**
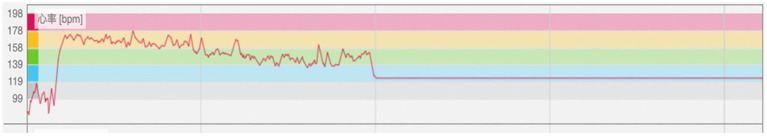
Statistical diagram of heart rate zones. Note: the second half of the training data is missing due to signal instability.

There were originally 292 sessions, of which 19 sessions were not participated by all athletes, so they were excluded, accounting for 6.5% of the total proportion (Figure 3).

#### 2.6.2 Data calculations

Regarding the sRPE calculation, we calculated the training load through psychological RPE. The calculation formula was as follows: Session RPE = training duration × RPE (Foster et al., 2001).

Regarding the TRIMP calculation, we used different intensity zones (heart rate zones) for the physiological load calculations. The calculation formula was determined as: TRIMP = training duration × training intensity coefficient (three levels; Lucía et al., 2002).

Lucia use individual physiological indicators measured in the laboratory to correspond to heart rate, so that heart rate values can correspond to specific physiological calibration points, such as aerobic threshold, anaerobic threshold, ventilation threshold, respiratory compensation point, etc. This method can better reflect athlete individualization without the need to collect blood lactate during training. This method has currently become the main calculation method for TRIMP. Therefore, this study adopts Lucia’s three-level method.

To avoid the influence of repeated measurements within individual sessions on correlation statistics, this study used the mean of all athlete-related parameters from each training session as independent sample data.

### 2.7 Statistical methods

The sample size of this study was calculated by Gpower. The statistical analysis was used to focus on the comparison of the data obtained after each training session using two different methods: session RPE and TRIMP.

SPSS26 software was used for all statistical analysis, and the Shapiro–Wilk test was used to test the normal distribution of the data. Normally distributed measurement data are expressed as mean ± standard deviation, and non-normally distributed measurement data as median data (maximum to minimum value mean).

When calculating the correlation, we selected Pearson’s correlation coefficient for the normal distribution data and Spearman’s coefficient for the samples that did not satisfy the normal distribution. *p* < 0.05 represented the division of the correlation strength with significant, respectively. The magnitude of r was used as follows: 0.1–0.29, weak correlation; 0.3–0.49, moderate correlation; 0.5–0.69, strong correlation; 0.7–0.89, very strong correlation; and 0.9–1.0, almost complete correlation (Hopkins et al., 1999).

For regression analysis, full-intensity training sessions, LIT, MIT, and HIT sessions were analyzed separately. Stata was used to test the differences in the slopes of the different regression equations (Chow, 1960), and *p* < 0.05 was regarded as the slope with structural differences.

## 3 Results

### 3.1 TRIMP and sRPE in different intensity training sessions

Using HIT, MIT, LIT, and the overall data, the correlation test between sRPE and TRIMP of the four training data samples was carried out, as well as a linear regression analysis. The results showed that there was a strong correlation between the overall data sample sRPE and TRIMP (*r* = 0.68, *p* < 0.05); sRPE in the LIT group was strongly correlated with TRIMP (*r* = 0.70, *p* < 0.05), and sRPE in the MIT group was moderately correlated with TRIMP (*r* = 0.46, *p* < 0.05); sRPE was moderately correlated with TRIMP in the HIT group (*r* = 0.31, *p* < 0.05; Table 5).

**Table 5:** Generalized linear mixed model with repeated-measures analysis: estimates of fixed effects with sRPE as dependent variable.

TRIMP	N	Estimate	SD	F	*p*	95%CI
Training type						
LIT	459	3.175	0.1501	447.7	<0.0001	2.880 to 3.470
MIT	37	3.274	1.038	9.141	0.0047	1.076 to 5.472
HIT	64	1.973	0.6908	8.154	0.0058	0.5917 to 3.354
Time point						
0-60 min	53	3.294	0.6255	27.73	<0.0001	2.038 to 4.550
60-120 min	240	2.895	0.2862	102.3	<0.0001	2.331 to 3.458
120-180 min	99	3.892	0.5488	50.28	<0.0001	2.802 to 4.981

CI, confidence interval; reference category; sRPE, session rating of perceived exertion; TRIMP, training impulse.

**Figure 4:**
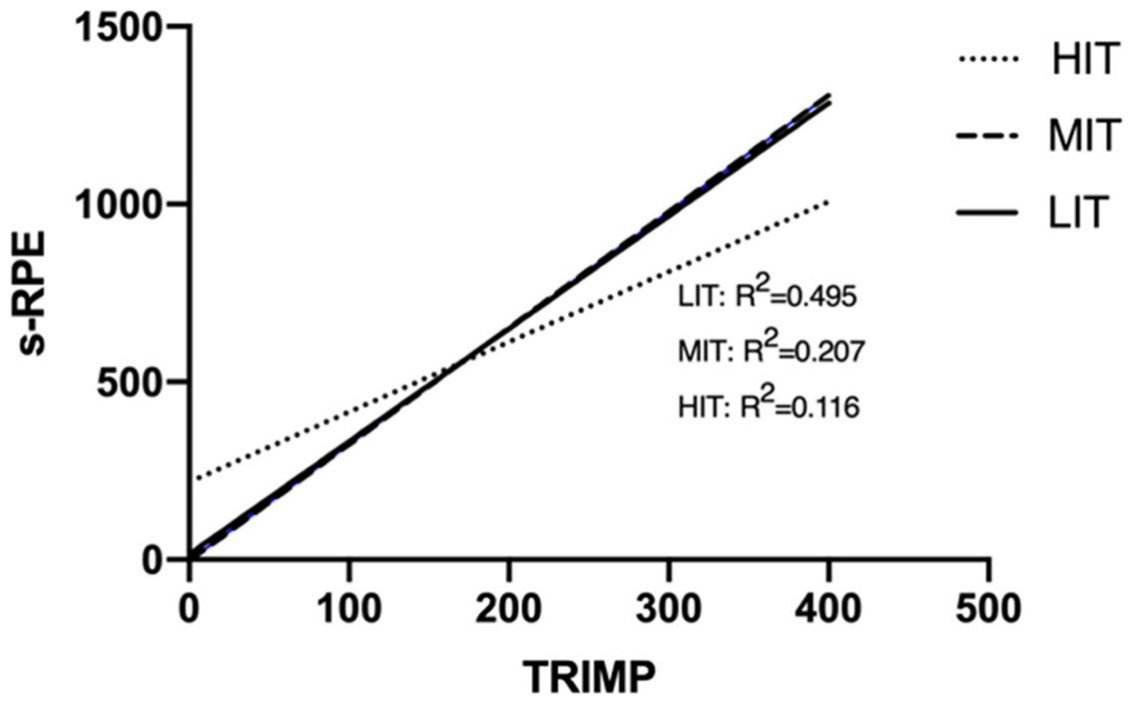
Linear regression graph of sRPE and TRIMP in different types of training sessions.

Using the three sets of training data of HIT, MIT, and LIT as samples, a linear regression was formed with sRPE as the dependent variable and TRIMP as the independent variable. The straight line after regression fitting is shown in Figure 4. After two-to-two testing of the three types of training courses by Chow test, the results show that there was no difference between MIT and LIT among the three regression lines, and there was a structural difference between HIT and the former two (*p* < 0.05).

### 3.2 TRIMP and sRPE of LIT sessions with different durations

**Figure 5:**
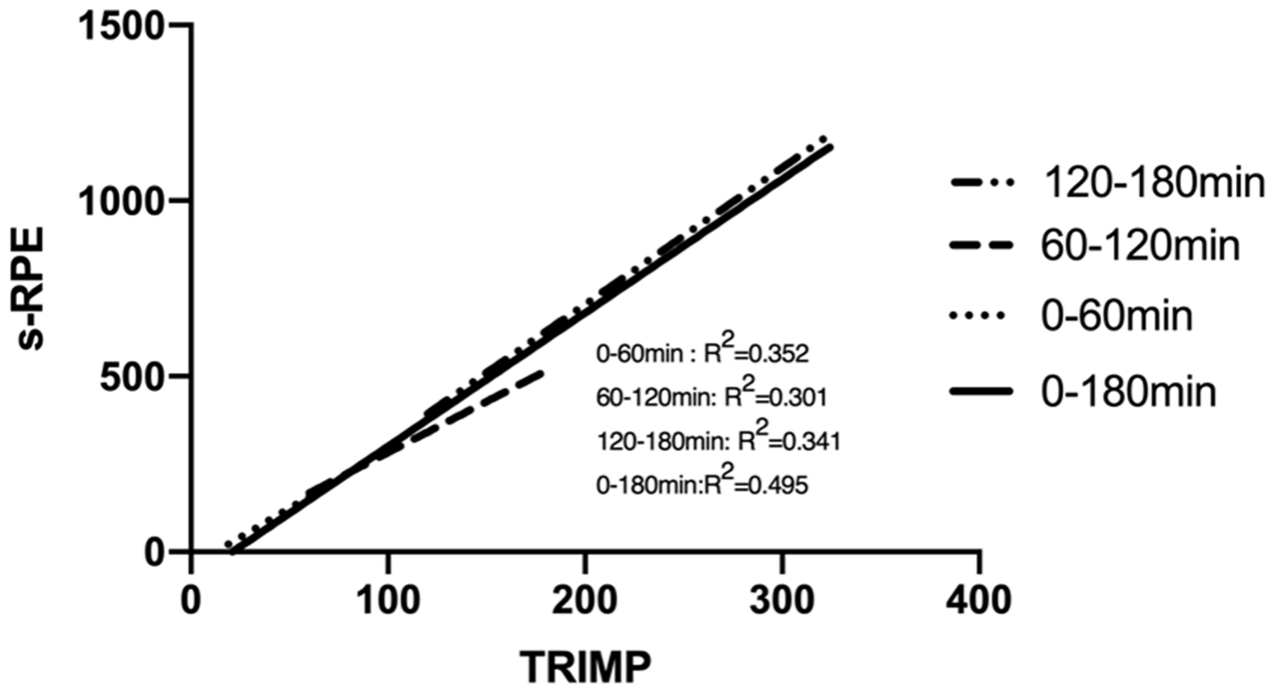
Linear regression plots of TRIMP and sRPE in LIT sessions at different durations.

The sRPE of the three time-length samples of 0–60 min, 60–120 min, and 120–180 min was used as the dependent variable, and TRIMP was used as the independent variable, to conduct a linear regression analysis. The results showed that the correlation between the independent variable and the dependent variable in the three sample “r” values showed a strong correlation (0–60 min: *r* = 0.70; 60–120 min: *r* = 0.67; 120–180 min: *r* = 0.69; *p* < 0.05). There was no significant difference between the slope of the regression line fitted by the three time-length samples and the slope of the overall period of 0–180 min (Figure 5). After the Chow test, it was found that the fitted curves of 0–60 min, 60–120 min, and 120–180 min had no structural difference between the training sessions and the fitting curves of TRIMP and sRPE in the training sessions of different training periods and the total training period of 0–180 min.

### 3.3 Influence of training duration calculation on sRPE and TRIMP relationship^1^

**Figure 6:**
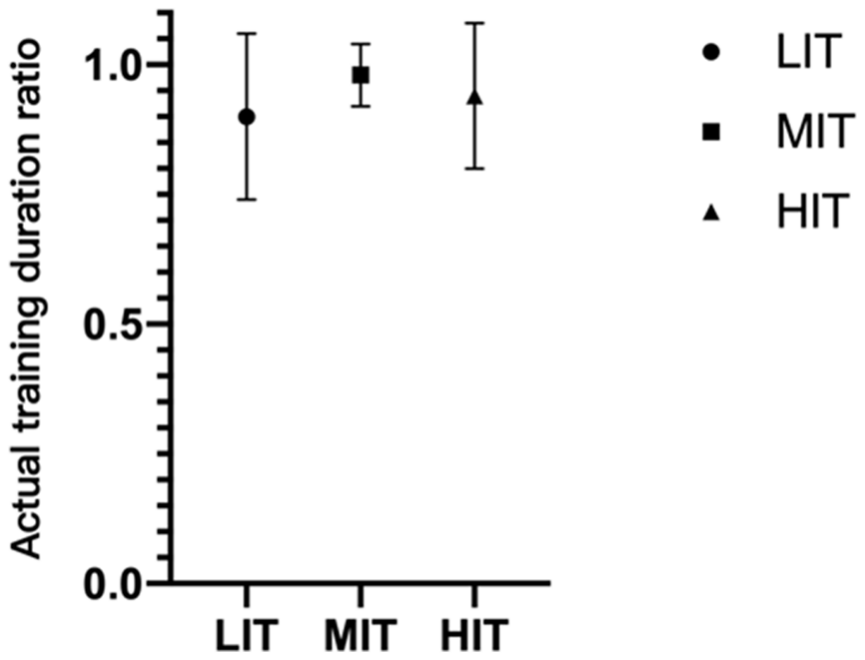
Actual training duration ratio of different training sessions.

The calculation of sRPE depends on the training duration of the training session, and ‘time in zone’ is a statistic of the actual training duration. This study found that this ratio varies across different intensity trainings (LIT: 89.7 ± 16.4%, MIT: 98.5 ± 6.2%, HIT was 94.4 ± 13.5%; Figure 6).

After adjusting the calculation method of training load from “planned training duration” to “actual training duration,” sRPE-TRIMP become more pronounced (Table 6), and the correlation coefficient “r” between sRPE and TRIMP in the HIT sessions is obvious.

**Table 6:** Influence of statistical methods of training duration on the sRPE-TRIMP correlation.

Training intensity	sRPE (planned training duration) and TRIMP / relevance	*p*	95%CI	sRPE (actual training duration) and TRIMP / relevance	*p*	95%CI
All intensity	*r* = 0.683	<0.0001	0.636 to 0.725	*r* = 0.764	<0.0001	0.727 to 0.796
LIT	*r* = 0.703	<0.0001	0.654 to 0.747	*r* = 0.791	<0.0001	0.754 to 0.823
MIT	*r* = 0.455	0.0047	0.154 to 0.679	*r* = 0.482	0.0025	0.187 to 0.697
HIT	*r* = 0.341	0.0058	0.104 to 0.541	*r* = 0.524	<0.0001	0.320 to 0.682

## 4 Discussion

There was a significant correlation between sRPE and TRIMP, and a strong correlation between sRPE and TRIMP in the LIT group. There was also a moderate relation between sRPE and TRIMP in the MIT and HIT groups. The difference in actual training duration between samples is the main reason for the difference in the application effect of sRPE, because the actual training duration ratio of LIT was 89.7 ± 16.4%; MIT, 98.5 ± 6.2%; and HIT, 94.4 ± 13.5%.

### 4.1 LRsRPE-TRIMP is affected by the type of training session

Researchers such as Foster support the fact that sRPE can be used as a load recording method for all types of courses, such as continuous and intermittent sessions (Foster et al., 2001). The validity of sRPE has been demonstrated and put to use in many different studies (Haddad et al., 2017). We confirmed the feasibility of converting sRPE to TRIMP in cross-country skiing, and there was a correlation between sRPE and TRIMP in all training session samples.

TRIMP is often used as a benchmark for validating sRPE due to the objectivity of heart rate data. In endurance activities, the variation in HR correlates more consistently with changes in VO_2_, ensuring the validity of TRIMP in endurance activities. However, most of the research objects that confirmed the significance of correlation between sRPE and TRIMP are present in ball sports, and the intensity of ball sports varies continuously (Haddad et al., 2017). The samples in this study are endurance training sessions, and the experiments confirmed the sRPE-TRIMP correlation significantly. This complements previous studies.

In addition, we verified that sRPE and TRIMP have strong correlation in LIT continuous training load, which is consistent with the results of the few related studies on endurance training (Manzi et al., 2015; Foster et al., 2017). Rodriguez, Manzi, and Haddad believe that sRPE has a moderate or strong correlation with TRIMP in endurance training (Rodríguez-Marroyo et al., 2012; Manzi et al., 2015). However, the above studies on endurance events did not distinguish the intensity, duration, and form of training sessions, which are essential factors affecting the reliable of sRPE-TRIMP correlation. This was further verified in our present study.

Previously, the academic community was divided on the reliability of sRPE-TRIMP correlation during interval training. Mingantl, Manzi, and Dellavalle have confirmed that sRPE strongly correlates with TRIMP in football, basketball, and rowing, respectively (Manzi et al., 2010; Minganti et al., 2011; DellaValle and Haas, 2013). Similar conclusions emerged for other interval training (Akubat et al., 2012; Rago et al., 2020). All of the above studies suggest that sRPE may be a more reliable measure of exercise intensity during interval exercise where both anaerobic and aerobic systems are properly activated (Bangsbo, 1994). In contrast, Haddad, and Chen et al. believe that sRPE-TRIMP correlation is less reliable during interval training or lower than that in continuous training endurance events (Michael et al., 2002; Impellizzeri et al., 2004; Haddad et al., 2011).

The results of this study show that sRPE-TRIMP correlation, especially in HIT is moderate (*r* = 0.31, *p* < 0.05); and related studies speculate that the sRPE-TRIMP correlation is slightly lower in interval items. A possible explanation for this is that the higher anaerobic energy ratio of interval training compared with continuous training may be the main reason for the reliability of sRPE-TRIMP correlation (Arazi et al., 2017). We speculate that, in addition to the impact of intensity, the uncertainty of intermittent time may be another important factor.

It should be noted that although HIT interval training has similarities with “intensity changes” in sports such as basketball, football, and ice hockey, the HIT sessions of endurance events such as cross-country skiing remains at aerobic intensity. The interval in the training session is “deliberate,” and the intensity change of the ball sports is not. Therefore, whether the viewpoint of sRPE used in interval training in this study can be transferred to ball sports needs further verification.

### 4.2 Consistency of LRsRPE-TRIMP is affected by the type of training session and not by time

Related research suggests that differences in the duration of training and the type of continuous versus intermittent training may lead to differences in the linear regression fit. The former conjectures that, with continuous training with constant intensity, as time goes on, the RPE may be overestimated and cause the overestimation of sRPE, which will lead to the difference of the linear regression equation (Green et al., 2009). In this study, the comparison of the linear regression equations of the three lengths of LIT (0–60 min, 60–120 min, and 120–180 min) found no structural differences among the three regression equations, which negated this conjecture. For the latter, this study compared the linear regression equations fitted by the three training samples of HIT, MIT, and LIT. There was no difference between MIT and LIT, but there were structural differences between HIT and the former two. This is different from the research results of Roos et al. (2018), which suggested that, under the condition of equal TRIMP, higher intensity training will lead to higher sRPE (Roos et al., 2018).

The results of this study, especially the comparison between LIT and MIT training sessions, refute this idea. In the MIT training course of cross-country skiing, the single-set training duration is generally more than 10 mins, and during training at this intensity, blood lactate levels consistently remain below 4 mmol/L. This kind of medium-intensity long-interval intensity is generally lower than the anaerobic threshold, the athletes are still primarily relying on aerobic energy metabolism, and it also has the characteristics of continuous training. We speculate that incomplete recovery in HIT may be an important reason for the difference between sRPE and TRIMP conversion compared with LIT and MIT. It can be seen from the research results that TRIMP = 170 is the threshold point, the sRPE of HIT is higher than that of MIT and LIT when it is lower than this value, and that the sRPE of HIT is lower when it is higher than this value. However, 90% of HIT training sessions are lower than TRIMP170 (about 127 min),^2^ which shows that HIT training sessions generally have higher TRIMP. We can guess that the discomfort caused by incomplete recovery in high-intensity training will gradually make athletes adapt as the training duration increases, so the slope of the HIT interval training session in Figure 4 is lower than that of the other two.

In summary, the difference between intermittent and continuous types may be the main factor affecting the consistency of LRsRPE-TRIMP. In contrast, training duration does not affect the consistency of LRsRPE-TRIMP in continuous training sessions.

### 4.3 Factors of the reliability of the sRPE-TRIMP correlation in XC skiing

#### 4.3.1 Training duration is not a factor affecting the sRPE-TRIMP correlation

The research results show that there is a highly significant correlation between sRPE and TRIMP in LIT training sessions, and the training duration (0–60 min, 60–120 min, 120–180 min) will not affect the reliability (*r* = 0.69, *r* = 0.65, *r* = 0.68; *p* < 0.05). In the calculation of sRPE, RPE represents training intensity and time represents training volume (Green et al., 2009). The training intensity of LIT remains stable, so RPE should also remain unchanged. The study by Green et al. regarding running trials of different durations (20, 30, and 40 min) showed that the impact of training duration on sRPE was weak (Green et al., 2009).

However, scholars such as Green hypothesize that extremely long training duration may affect the sRPE-TRIMP correlation. For example, under conditions of insufficient recovery from competition or training, under conditions of suboptimal glycogen recovery, or when exercise is long enough (at least 90 min), the depletion of muscle or liver glycogen is sufficient to have an effect on RPE (increased central motor command activity, thereby increasing the perception of fatigue), and duration may become a mediator variable with a substantial impact on RPE (Green et al., 2009).

The correlation analysis between RPE and duration using LIT training samples in this study showed that RPE was weakly correlated with training duration in LIT training, while there was no correlation in MIT and HIT (LIT: *r* = 0.19, *p* < 0.05; MIT: *r* = 0.14, *p* > 0.05; HIT: *r* = 0.02, *p* > 0.05). This suggests that RPE may not be affected as training duration increases. This result extended the influence of Green et al. on the validity of training duration on RPE measurements from 40 min to 180 min (Green et al., 2009). At the same time, it also refuted the conjecture that the extremely long training duration may affect the sRPE-TRIMP correlation.

#### 4.3.2 Training duration deficit^3^ is an essential factor affecting the sRPE-TRIMP correlation

**Figure 7:**
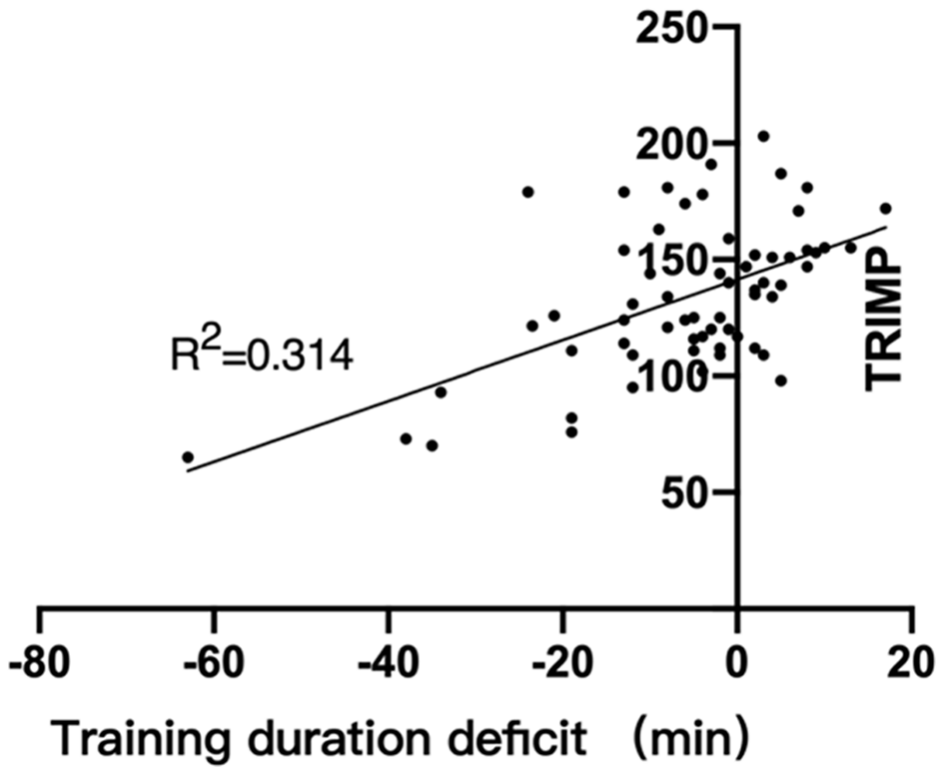
Relationship between training duration deficit and TRIMP.

The results of this study show that there is a discrepancy between the actual training duration and the planned training duration, the difference between these two is referred to as the ‘Training duration deficit’. When using the actual training duration to calculate the sRPE improves the sRPE-TRIMP correlation in various training sessions. In addition, the sRPE-TRIMP correlation was much lower than that of LIT in HIT and MIT training sessions. After using the actual training duration to calculate sRPE, the “r” value of the correlation between sRPE and TRIMP in the HIT class increased from 0.34 to 0.52, and the correlation improvement was higher than that of LIT and MIT. Through further data analysis, it was found that there is a very significant positive correlation between the training duration deficit and the TL (*r* = 0.56, *p* < 0.05); that is, the more the actual training duration is lower than the planned training duration, the lower the actual TL. It can be understood that endurance athletes are affected by certain factors that will cause excess fatigue, and that this factor may directly or indirectly cause athletes to fail to complete the planned training volume through excess fatigue. This phenomenon is most obvious in HIT training sessions (Figure 7).

From empirical observations, scholars such as Foster point out that one of the potential reasons for the high incidence of adverse results in training is the lack of correspondence between the plans developed by the coach and the plans executed by the athletes (Foster et al., 2001). In this study, we found that, even if the coaches make plans for the athletes according to the unified training duration and intensity, there will still be differences in the understanding and implementation of the training plan by the athletes. We believe that some factors other than TL affect both the athlete’s sRPE and the actual training duration of the athlete, and indirectly have a more significant impact on the calculation of sRPE. These factors may include factors such as training session settings, fatigue accumulation, and emotions.

1 Training session setting: The arrangement of the training session content needs to be adapted to the athletes. Usually, in the arrangement of HIT training, it is hoped that athletes will gradually find the “training state” in the first few sets of intensity training. This “training state” can activate the heart and lungs, and mobilize the respiratory and circulatory systems better. At this time, when the muscles are relatively relaxed, a higher aerobic intensity can be achieved during training, resulting in better training adaptation. The arrangement of the content of the training session will affect the pace at which athletes adapt to the intensity. When athletes increase intensity in a gradual manner during training, subjective fatigue may be reduced. However, cardiopulmonary-based fatigue of the respiratory system differs from muscle fatigue (Los Arcos et al., 2016). Another situation that may occur when the training settings include some sprint sets, the muscles soon fatigued in advance and the heart and lungs are not mobilized, resulting in excessive anaerobic energy being mobilized. This will make the athlete’s muscles stiff in advance, and the subjective fatigue feeling value will be higher. The record of TRIMP is based on the response of the aerobic system energy supply to the load based on the heart rate, and cannot reflect the work tasks of the anaerobic energy supply system. Therefore, when monitoring the training load, the TRIMP value based on the heart rate is low, and the subjective fatigue feeling RPE value may be high. The same phenomenon was also mentioned in another study.

One studies have proposed the use of plotting heart rate and velocity sequences (2-dimensional kernel density estimation-KDE) to couple internal and external loads, and found that ‘all training session types differed when comparing KDEs for heart rate and velocity’ (both *p* < 0.001; Van der Zwaard et al., 2023). The speed skating athletes investigated in this study applied various types of variable intensity training such as Extensive endurance, Extensive interval, Intensive endurance, Tempo, Sprint, etc. Differences in heart rate at the same velocity are related to oxidative metabolism, as shorter-duration high-speed training cannot elicit a matched high heart rate, but can result in higher perceived exertion (RPE). However, unlike speed skating, cross-country skiing involves a more upright skiing posture and places greater emphasis on stimulating VO2 max during HIT, thus resulting in less variability in HIT training session types. Therefore, applying the same methods to study speed skating may lead to less significant sRPE-TRIMP correlation in HIT training.

2 Fatigue accumulation: Training under fatigue accumulation will affect athletes’ training experience for HIT. High-intensity training stimulates the athlete’s oxygen uptake capacity as well as the neuromuscular system. In this type of training, athletes need to have a good training state and the ability to impact the circulatory system and neuromuscular system through high-intensity intervals. Low-intensity training is used as the “base” to improve muscle capillarization, accumulate the total amount of training, and recover from high-intensity training to transition to the next high-intensity training.

In order to ensure the quality of the high-intensity training session, it needs to be fully recovered from the previous low-intensity training. And high-intensity training sessions are more susceptible to fatigue accumulation in the previous 2 days than low-intensity training sessions. In the case of accumulated fatigue, athletes may “excessively work hard” to produce excessive RPE in order to achieve high training intensity.

There is not much difference in training volume between full exhaustion and incomplete exhaustion, and the intensity is only to maintain the previous intensity. This kind of “all-out” training has a high impact on RPE. Training under this kind of fatigue may keep the athlete in an “all-out” state, which will make the athlete more fatigued and even affect the recovery speed.

3 “Stress” and “Emotions”: Stress and negative emotions may be the cause of premature fatigue. “Stress” and “emotion” represent mental structures related to fatigue (Fessi and Moalla, 2018; Draper et al., 2021; Salagaras et al., 2021). Athletes may feel fatigued when they are stressed by the content of the training session, which affects the RPE score. Generally speaking, the stimulation of high-intensity training sessions will make athletes feel stressed, while low-intensity training sessions may cause less stress and resistance in athletes.

Both the central nervous system and the peripheral nervous system may be factors that affect muscle fatigue (Wan et al., 2017; Behm et al., 2021). Central fatigue is caused by a reduction in neural drive, which leads to a reduction in the number of motor units recruited and their so-called “firing” (Boyas and Guével, 2011; González-Izal et al., 2012).

The reason for the decrease in neural drive is currently an explanation that the ratio of serotonin to dopamine increases. Serotonin, which is associated with the central nervous system, is associated with increased dopamine and “stress” and “mood,” which may contribute to earlier feelings of muscle fatigue.

However, the above conjectures about the related factors that affect the difference in the actual training duration of athletes, and then affect the calculation of LRsRPE-TRIMP, need to be confirmed by further research.

## 5 Conclusion and recommendation

### 5.1 Conclusion

1. The LRsRPE-TRIMP is more significant in LIT compared to other intensity type, and the significance in HIT is the lowest.
2. The difference in the duration of LIT classes does not affect the consistency of sRPE in characterizing its training load.
3. The difference between actual training duration and planned training duration (training duration deficit) directly impact the significance of the LRsRPE-TRIMP, which is most evident in HIT training courses.

### 5.2 Recommendation

1. The use of sRPE to convert training load to supplement missing data in off-road skiing training is a reliable means, but for specific use, different intensity training courses can refer to the different standards.
2. The sRPE-TRIMP correlation in HIT may be influenced by factors such as fatigue load accumulation and training course content arrangement. Coaches should strive to maintain consistent intensity course settings and requirements, which can form a more consistent relationship between the sRPE of intensity courses and training load.
3. The conclusion of this study is limited to cross-country skiing, and similar conclusions may arise due to the different exercise modes of other endurance events compared to cross-country skiing, but separate research is needed.

## Data availability statement

The datasets presented in this study can be found in online repositories. The names of the repository/repositories and accession number(s) can be found in the article/supplementary material.

## Ethics statement

The studies involving humans were approved by Ethics Committee for Sports Science Experiments at Beijing Sport University. The studies were conducted in accordance with the local legislation and institutional requirements. Written informed consent for participation provided by the participants.

## Author contributions

SY: Writing – original draft. QM: Writing – review & editing. ZQ: Writing – review & editing. YY: Conceptualization, Methodology, Writing – review & editing.

## Footnotes

^1^The planned training duration is the overall arrangement of the training session by the coach when making the plan, including the time length of warm-up, training, interval, and cool-down; the actual training duration refers to the heart rate intensity exceeding the training duration in all links of training. The total length of time for the minimum threshold.

^2^The training duration of the HIT training course and TRIMP establish a linear regression: TRIMP = 1.145 × duration +24.5, when TRIMP = 170, the training duration is about 127 min.

^3^Training duration deficit = actual training duration–planned training duration.
